# Toward the Development of the Trojan Female Technique in Pest Insects: Male‐Specific Influence of Mitochondrial Haplotype on Reproductive Output in the Seed Beetle *Acanthoscelides obtectus*

**DOI:** 10.1111/eva.70065

**Published:** 2024-12-26

**Authors:** Lea Vlajnić, Uroš Savković, Jelena Jović, Sanja Budečević, Biljana Stojković, Mirko Đorđević

**Affiliations:** ^1^ Faculty of Biology, Institute of Zoology University of Belgrade Belgrade Serbia; ^2^ Department of Evolutionary Biology, Institute for Biological Research “Siniša Stanković”‐National Institute of the Republic of Serbia University of Belgrade Belgrade Serbia; ^3^ Department of Plant Pests Institute for Plant Protection and Environment Zemun Serbia

**Keywords:** longevity, male infertility, mitonuclear interactions, Mother's Curse, pest control, sex‐specific effects

## Abstract

Biocontrol techniques that impair reproductive capacity of insect pests provide opportunities to control the dynamics of their populations while minimizing collateral damage to non‐target species and the environment. The Trojan Female Technique, or TFT, is a method of the trans‐generational fertility‐based population control through the release of females that carry mitochondrial DNA mutations that negatively affect male, but not female, reproductive output. TFT is based on the evolutionary hypothesis that, due to maternal inheritance of mitochondria, mutations which are beneficial or neutral in females but harmful in males can accumulate in the mitochondrial genome without selection acting against them. Although TFT has been theoretically substantiated, empirical work to date has focused only on *Drosophila melanogaster* populations, while the existence of male‐biased mutations and the TFT approach in economically important pest species remain unexplored. Here, we examined the sex‐specific effects of three distinct and naturally occurring mitochondrial haplotypes (MG1a, MG1d, and MG3b) on several reproductive and life history traits in the seed beetle *Acanthoscelides obtectus* . Our results revealed that males harboring the MG3b mitotype exhibited lower early fecundity and fertility, while there were no effects on females or longevity in either sex. Our experiments provide support for the existence of the mitochondrial variant that specifically impairs male reproductive output in pest insects. These results can be harnessed to further develop TFT as a novel form of biocontrol with broad applicability to economic pests and disease vector insects.

## 1 Introduction

Mitochondrial and nuclear genes coordinate closely to regulate one of the most important biological functions of life—energy production via oxidative phosphorylation (Rand, Haney, and Fry 2004; Wolff et al. 2014). Even a minor incompatibility in the mitonuclear interaction can lead to biochemical inefficiencies of OXPHOS and an increase in oxidative stress. Therefore, strong selection pressure ensures the maintenance of the optimal mitonuclear allele combination throughout the evolutionary trajectory of each population in its environmental context (Stojković and Đorđević 2017). Thus, at its core, mitochondrial functionality hinges on the cooperation and coevolution of the mitochondrial (mtDNA) and nuclear (nDNA) genomes. Several empirical studies have shown that mitonuclear interactions are associated with the expression of various physiological and life history traits through their efficiency in regulating energy allocation (Arnqvist et al. 2010; Đorđević et al. 2015, 2017; Immonen et al. 2016; Mossman et al. 2016; Vaught and Dowling 2018).

Despite this cooperative relationship between the two genomes, evolutionary theory also predicts the existence of a sex‐specific mitonuclear conflict due to sexual asymmetry in mitochondrial transmission (Partridge and Hurst 1998). Namely, natural selection can only act directly on the non‐neutral mtDNA polymorphisms through the female lineage since mitochondria are inherited from the mothers. As a result, the mtDNA mutations, which are male harming but neutral or beneficial to females, are not subject to negative selection. In other words, the male‐biased mtDNA mutations will evade selection and accumulate within the mitochondrial genomes, leading to an accumulation of male‐detrimental mtDNA mutations (Frank and Hurst 1996; Innocenti, Morrow, and Dowling 2011; Smith and Connallon 2017). This form of intralocus sexual conflict has been termed Mother's Curse hypothesis (Gemmell, Metcalf, and Allendorf 2004; Havird et al. 2019) and mtDNA mutations that reduce male fitness while being benign or beneficial to females are named Mother's Curse (MC) mutations (Camus and Dowling 2018; Dowling and Adrian 2019).

However, the Mother's Curse Hypothesis predicts that the accumulation of the male‐detriment MC variants will invoke strong selective pressure on the nuclear genome to evolve a set of restorer alleles that compensate for the reduction in male fitness (Connallon et al. 2018; Munasinghe, Haller, and Clark 2022). The evolutionary dynamics of MC mutations and compensatory nuclear counter‐adaptations represent a conflict‐driven molecular arms race between the mitochondrial and nuclear genomes (Touzet and Budar 2004). Therefore, Mother's Curse mutations can segregate cryptically in populations as they are masked by nuclear alleles that restore male function. The theory suggests that in order to detect the male‐harming mitochondrial variants within a population it is necessary to disrupt the evolved mitonuclear interactions, which will lead to the negative epistasis for fitness in males rather than in females (Dowling and Adrian 2019). Few studies have provided evidence for mitonuclear coevolution based on nuclear rescuing modifiers, both in angiosperms and metazoans (Budar, Touzet, and De Paepe 2003; Fujii, Bond, and Small 2011; Patel et al. 2016; Smith, Turbill, and Suchentrunk 2010; Yee, Sutton, and Dowling 2013). In addition, many studies confirmed the male bias in the influence of mitochondrial genetic variation and mitochondrial bioenergetics, metabolic parameters, and all upstream life history traits (Aw et al. 2017; Camus, Clancy, and Dowling 2012; Camus et al. 2015; Drummond, Short, and Clancy 2019; Đorđević et al. 2015, 2017; Nagarajan‐Radha et al. 2020; Wolff et al. 2016; Yee, Sutton, and Dowling 2013). This is particularly true for reproduction, a metabolically demanding trait that displays sexual dimorphism (Dowling, Nowostawski, and Arnqvist 2007; Immonen et al. 2016). Consequently, one of the most commonly observed Mother's Curse phenotypes is male subfertility (Nakada et al. 2006; Vaught and Dowling 2018).

The existence of the Mother's Curse effect has been recognized as an opportunity for the development of a genetic biocontrol approach termed the sterile insect technique (SIT). SIT introduces large quantities of irradiated males into target pest population in each generation (Dyck, Hendrichs, and Robinson 2021). Mating between sterile males and wild females produces less progeny and leads to a reduction in population size in the next generations. Repeated releases result in a population being greatly suppressed or even eradicated. Although SIT is species‐specific and has been used to control various pest insects (Bourtzis et al. 2016; Pereira et al. 2013; Tabashnik et al. 2010), several drawbacks limit its long‐term sustainability (Teem et al. 2020). First, it requires continuous production and frequent releases of large numbers of irradiated individuals, which is time‐consuming and costly. Second, it is more effective when only males are released (Alphey and Bonsall 2018). Finally, irradiation can compromise sexual performance and survival of SIT males, making them less competitive in mating than their wild counterparts (Zhang et al. 2016). The technique would be much improved if sterile males could be produced continually within the populations targeted for control, without the need for sex sorting, manual irradiations, and introductions in each generation. The transgenerational heritability of male sterility could be employed by some form of genome editing (Alphey and Bonsall 2018; Häcker, Bourtzis, and Schetelig 2021). However, the release of genetically engineered animals into nature raises ethical, safety, and regulatory concerns.

Recently, a heritable form of SIT has been proposed based on naturally occurring Mother's Curse mtDNA mutations and referred to as the Trojan Female Technique (TFT) (Gemmell et al. 2013). Theoretically, females carrying MC mutations, and their healthy female descendants, could continuously produce subfertile males that sire fewer offspring than their wild‐type counterparts over multiple generations. Therefore, if females with such an MC mutation, a candidate mutation for TFT, could be identified, harnessed, and released, this could provide a self‐perpetual population suppression of pests over multiple generations after a single release of Trojan females into the wild. The applicability of TFT for pest control has been theoretically substantiated (Gemmell et al. 2013), but empirical attention has focused only on a particular mitochondrial haplotype of the fruit fly ( *Drosophila melanogaster* ) termed Brownsville (BRO) (Dowling, Tompkins, and Gemmell 2015; Wolff et al. 2016, 2017). This haplotype harbors a point mutation in the mitochondrial cytochrome b gene that is associated with complex and sex‐specific effects, most notably including depressed fertility and lifespan in males but not in females (Camus, Clancy, and Dowling 2012; Yee, Sutton, and Dowling 2013). Although the TFT is a promising non‐transgenic, self‐sustaining, trans‐generational biocontrol of target pest species, its practicability has not yet been investigated in an invasive insect species affecting agriculture.

In the present study, our overall aim was to identify potential TFT candidate in the pest insect *Acanthoscelides obtectus* by testing two main criteria: (i) MC mutations must reduce male, but not female, reproductive output and (ii) male‐fertility‐reducing mutations must have limited or no positive pleiotropy in other life history traits between the sexes. Assessing the effects of the MC mutation on quality parameters such as longevity of TFT females and males is essential because the effectiveness of TFT is associated with the ability of TFT beetles to compete with their wild counterparts for mating. In other words, the fitness costs of TFT candidate mutations are a key determinant for successful biocontrol. Here, we examine several reproductive and life history effects of three distinct and naturally occurring mitochondrial haplotypes (mitotypes) placed alongside the same outbred nuclear background in each sex of *A. obtectus* .

## 2 Materials and Methods

### 2.1 Study Species

The seed beetle *Acanthoscelides obtectus* (Say) (*Coleoptera*: *Chrysomelidae*: *Bruchine*) is an oligophagous pest of leguminous crops. The common bean, *Phaseolus vulgaris* L. is its primary host plant. Bean infestation starts in the fields by female oviposition into pods and then spreads in storages causing rapid destruction of bean seeds in subsequent generations (Schmale et al. 2002). For this reason, seed beetles are well adapted to storage conditions, and the laboratory environment provides a close approximation of their natural environment. The entire larval and pupal development of this species takes place within a seed. The adult beetles emerge from the seeds and are ready to mate after a few hours.

The *Acanthoscelides obtectus* beetles used in this study originated from the L laboratory population (Đorđević et al. 2015) which has been maintained on common bean seeds for more than 250 generations. All experiments reported here were performed in a dark incubator at 30°C. As the adults are facultatively aphagous, that is, they obtain all resources necessary for survival and reproduction during the larval stage, no food or water was offered to the adults. To avoid any possible infestation with pests, the seeds were frozen at −20°C for 24 h before use.

### 2.2 Construction of Mitochondrial Lines

To characterize the effect of the mitochondrial genome on reproductive output and longevity of the bean beetles, we created distinct mitochondrial (mt) lines by placing different naturally occurring mitochondrial haplotypes sourced from laboratory populations selected for late (L) reproduction alongside an outbred L nuclear background. A detailed description of the construction of the introgression mt lines can be found in Đorđević et al. (2015). Briefly, we mixed approximately 300 beetles from each of the four replicate populations from L selection line and propagated the four‐way cross L line for two generations [(L1 × L2) × (L3 × L4), where the subscript number refers to the specific replicate population]. We randomly collected 20 virgin one‐day‐old females from the four‐way crossed L line to perform the initial backcrossing. These females represented the founders of our mitochondrial lines (carrying a distinct mtDNA haplotype). Each female was placed in a jar with five randomly chosen one‐day‐old L males from the four‐way crosses. In the next generation, five randomly selected virgin F_1_ daughters of each haplotype were again backcrossed to five randomly chosen virgin one‐day‐old L males. This backcrossing scheme was repeated for 16 generations, replacing 99.998% of the original nuclear genome. The four‐way outcrossing of replicate populations and further mating of experimental females with males originated from this large outbred population allowed us to express each mt haplotype alongside a representative pool of nuclear variation (Dowling and Adrian 2019). Furthermore, this procedure diminished any epistatic interaction and linkage among genes that could have occurred during the long‐term selection. As a result of this backcrossing procedure, 20 distinct experimental mt lines were obtained.

### 2.3 mtDNA Genotyping

We screened for mitotype variability in the laboratory populations of *A. obtectus* within the obtained 20 mt lines before setting up experiments and defining an experimental design to test the MC effect that could serve as TFT. We searched for nonsynonymous substitutions in eight mtDNA genes that code for proteins that are involved in different electron‐transport‐chain (ETC) complexes (Table 1). For these tests, females from the 20 distinct L line populations were used, and the differences between them were compared to *A. obtectus* mitochondrial genomes that had previously been published (Sayadi et al. 2017; Yao, Yang, and Dai 2017). For each of the genes, we designed primers following conserved positions recognized by Simon et al. (2006) and matching the primer sequence to the *A. obtectus* mitogenome (Table S1). We first sequenced the eight selected protein‐coding mtDNA genes (Table 1), and based on the highest differences in resulting amino acid substitutions, we chose three mitotypes for use in experiments testing reproductive and life history effects when placed alongside the same outbred nuclear background. Later, we complemented results on genetic and resulting protein variability with the addition of five remaining mtDNA protein‐coding genes for the selected mitotypes tested for TFT, that is, MG1a, MG1d, and MG3b (see Table 2). Thus, we obtained sequences of all 13 protein‐coding mtDNA genes with a total length of 6679 bp (Table S1).

**TABLE 1:** Diversity of mitotypes identified within *Acanthoscelides obtectus* experimental L line by genotyping eight protein coding mtDNA genes screened for variability.

No.	Mitochondrial genotype	MLST code							
		*cox1*	*cox2*	*cox3*	*nad4*	*nad4L*	*nad6*	*cytb*	*nad1*
1	MG1a	1	1	2	2	2	2	2	2
2	MG1b	1	1	1	2	2	2	2	2
3	MG1c	1	1	1	2	2	1	2	2
4	MG1d	1	1	1	2	2	2	2	1
5	MG2a	2	1	2	2	2	2	2	2
6	MG2b	2	1	2	1	2	2	2	2
7	MG3a	3	2	3	3	3	3	3	3
8	MG3b	3	3	3	3	1	3	3	3

*Note:* Each number in the column for that gene denotes a distinct gene haplotype.

Abbreviation: MLST = multi locus sequence typing.

**TABLE 2:** Amino acid (aa) substitutions identified within screened mtDNA genes of *Acanthoscelides obtectus* experimental L line mitotypes selected for TFT testing.

Mitotype	MLST code	Amino acid (aa) position of substitutions from the start of the mtDNA genes^a^														
		*cox1*	*cox2*	*cox3*	*nad4*	*nad4L*	*nad6*	*cytb*	*nad1*	*nad3*	*nad5*	*atp8*	*atp6*		*nad2*	
			34 aa	169 aa				334 aa	171 aa			20 aa	45 aa	64 aa	73 aa	138 aa
MG1a	1122222211111	/	Ile	Ile	/	/	/	Ile	Asp	/	/	Phe	Gln	Ser	Ile	Val
MG1d	1112222111121	/	Ile	Thr	/	/	/	Ile	Asn	/	/	Phe	Arg	Ser	Ile	Val
MG3b	3333133311232	/	**Val**	Ile	/	/	/	**Val**	Asp	/	/	**Tyr**	**Leu**	**Lys**	**Thr**	**Met**

*Note:* Unique substitutions that were identified in mitotype experimentally evidenced to have Mother's Curse (MC) effect are marked in bold.

^a^ Absence of amino acid substitutions in the coding sequence of analyzed mt genes is marked with “/”.

Amplifications of the protein‐coding mtDNA genes were performed in the 12 independent polymerase chain reactions (PCR) using gene or gene region‐specific primers as detailed in Table S1. Amplification reactions were performed in a 20 μL final reaction volume containing a High Yield Reaction Buffer A with 1.5 mM MgCl_2_ (1×), an additional 1.5 mM MgCl_2_, 0.6 mM of each dNTP, 0.5 μM of each primer, 1 U of FastGene Taq DNA polymerase (NIPPON Genetics Europe), and 1 μL of DNA extract. The concentration of additional MgCl_2_ was different for the amplification of *cox2* and *nad4l*‐*nad6* (3.5 mM), *atp8*‐*atp6* (2.25 mM), and *cox1* (1 mM). Amplification of the *nad2*, *nad3*, and *nad5* genes was done using a High Sensitive Reaction Buffer B, instead of Buffer A. PCRs were carried out in a Mastercycler ep gradient S (Eppendorf, Hamburg, Germany) or GeneExplorer BYQ6631E (Hangzhou Bioer Technology, China) applying the following thermal protocol: 95°C for 5 min (initial denaturation), and 35 cycles at 95°C for 1 min, 1 min at 54°C (annealing), 72°C for 1 min 30 s, and a final extension at 72°C for 7 min. For the amplification of the *nad2*, *nad3*, *nad5*, and *atp8*‐*atp6* gene regions, the annealing temperature was 50°C, while for *nad4l*‐*nad6*, it was 52°C.

All sequence reads were obtained using the Sanger sequencing methodology by a commercial service (Macrogen Europe, Amsterdam, Netherlands). Sequences were edited using FinchTV v.1.4.0 (http://www.geospiza.com) and aligned using Clustal W (Thompson, Higgins, and Gibson 1994) within the MEGA v.5.2 software (Tamura et al. 2011). The nucleotide sequences were submitted to NCBI GenBank under accession numbers PP556977‐9 for *cox1*, PP756514‐6 for *cox2*, PP756499‐501 for *cox3*, PP756502‐4 for *nad4*, PP756505‐7 for *cytb*, PP756508‐10 for *nad1*, PP756511‐3 for *nad4l* and *nad6*, PP756517‐9 for *nad3*, PP756520‐2 for *nad5*, PP756523‐5 for *atp8* and *atp6*, and PP993233‐5 for *nad2*.

### 2.4 Experimental Design

**FIGURE 1:**
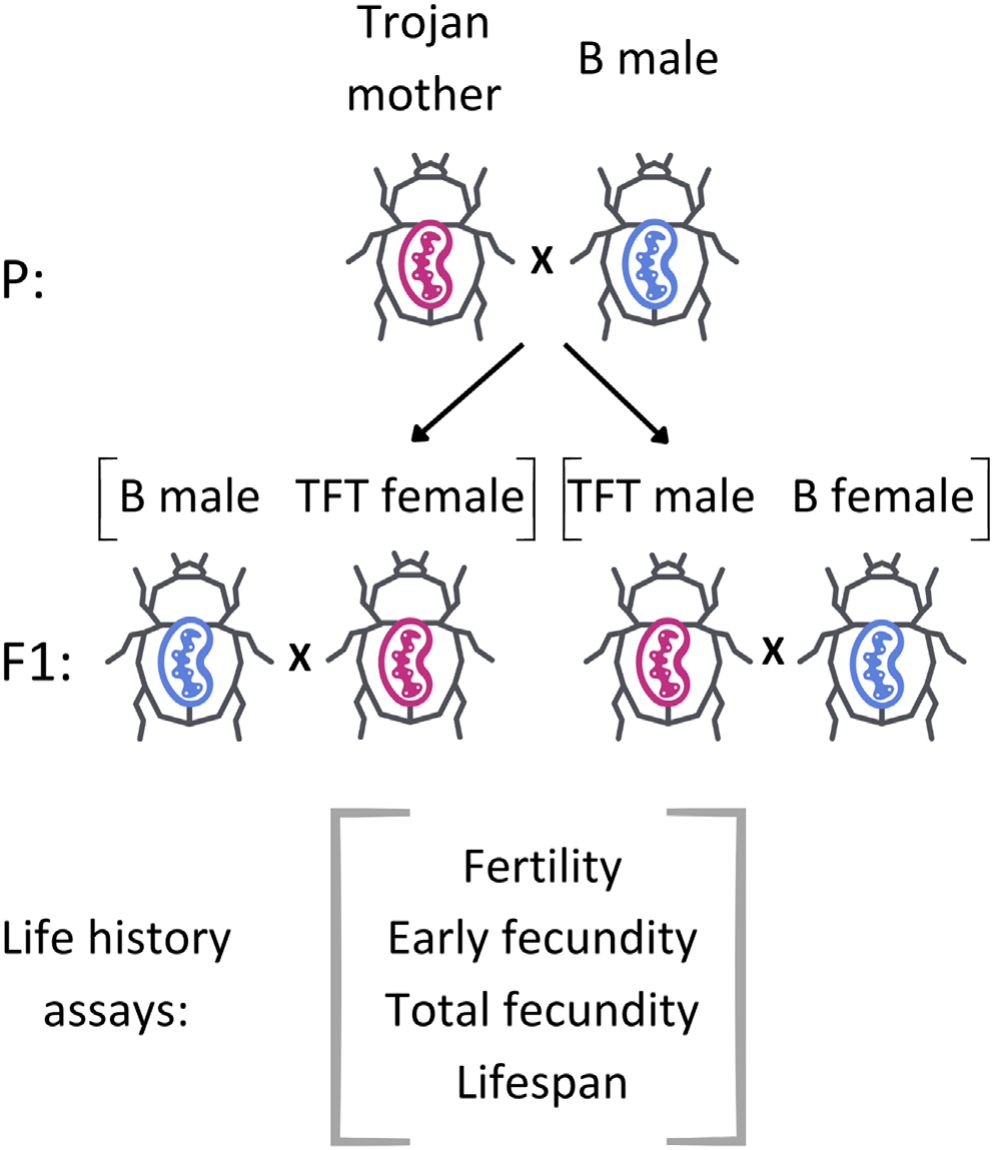
Schematic diagram of the experimental design used to assess sex‐specific effects of mitotypes on life history traits in *Acanthoscelides obtectus* . In the parental generation (P), crosses were conducted between virgin females (Trojan mother) possessing one of the three mtDNA haplotypes (MG1a, MG1d, or MG3b; red mitochondria) and virgin males of the outbred Base population (B male; blue mitochondria). In the F_1_ generation, TFT females and males (harboring red mitochondria inherited from their Trojan mother) were collected as virgins, and their reproductive performance and lifespan were recorded after mating with the reference virgin B females and males.

In order to detect the candidate TFT mutations, it is necessary to unmask the effects of mtDNA variation by placing mtDNA haplotypes alongside a novel nuclear background (i.e., to disrupt the mitonuclear adaptations (see above)). Accordingly, we mated 30 virgin females (Trojan mothers) of the specific mitochondrial lines (MG1a, MG1d, or MG3b) with the same number of randomly selected virgin males from another laboratory population, named Base (B) population (Figure 1). The B population was chosen because it exhibits high genome‐wide heterozygosity and maximizes the nuclear allelic variation that mitochondrial haplotypes were expressed against. This provided robustness for testing of the sex‐specific effects and thus the general utility of the candidate TFT mutation in the novel nuclear environment. We used the F_1_ TFT females and males as focal individuals in all the life history experimental assays, assessing fertility, early and total fecundity, and longevity. In sum, the focal F_1_ TFT females and males had one of the three mtDNA haplotypes (inherited from the Trojan mother), expressed alongside an outbred L haploid nuclear copy (also inherited from the Trojan mother) and a paternally‐contributed haploid nuclear copy derived from the outbred B population. The F_1_ TFT females and males were collected as virgins, and their reproductive performance and longevity were recorded after mating with the reference females and males (reference individuals were sourced as one‐day‐old virgins from an outbred B population).

The three haplotypes that we used in our experiments were present in more than two independent mt lines (they started with a different founder female, but they carry the same mtDNA haplotype). Specifically, each haplotype was represented with two independent lines, in total 6 mt lines. We named these 6 groups of beetles as “mt replicas.” The use of independent lines of focal mitotypes allowed us to statistically partition the true mitochondrial effects from nuclear effects (from cryptic nuclear variance that may have accumulated within the independent lines) and other sources of variation (Dowling and Adrian 2019).

### 2.5 Experimental Procedures

#### 2.5.1 Fertility

Upon emergence from each mt line, one virgin focal F_1_ TFT female was paired with a reference B male. Each pair was placed in a Petri dish containing bean seeds. Sixty individual pairs per mt replica were made. The same procedure was done for the F_1_ TFT males that were paired with B females. In total, we created 720 pairs (6 mt replicas × 60 pairs × 2 sexes = 720 pairs). Pairs were left in the incubator during their whole lifespan. Fertility was scored as the number of offspring from each Petri dish associated with each pair.

#### 2.5.2 TFT Female Reproductive Output and Lifespan

To characterize the pattern of fecundity and senescence in TFT females, the number of eggs and longevity of focal females were recorded by mating them with reference B males. Specifically, one‐day‐old virgin F_1_ TFT females from each mt replicate were randomly assigned to virgin B males. Around 60 pairs for each of the 6 mt replicas (in final sum 350 pairs) were made and placed in 35‐mm Petri dishes with bean seeds, where they were allowed to mate and lay eggs throughout their lives. The number of eggs and the longevity status of the beetles were recorded daily. Adult lifespan was measured as the time from the adult emergence until death. Total fecundity was estimated for each female as the total number of eggs laid during her lifetime. Early fecundity was measured as the absolute number of eggs laid within the first 2 days of age. Adults were weighed upon emergence to the nearest 0.0001 g to account for the effect of weight on fecundity and longevity.

#### 2.5.3 TFT Males Reproductive Output and Lifespan

To estimate the effect of mtDNA haplotypes on the reproductive success and lifespan of TFT males, a single freshly emerged F_1_ male collected from each mitochondrial replica was mated with single virgin B female. In this experiment, around 60 pairs per 6 mt replicas (329 in total) were placed in Petri dishes with bean seeds and their lifespan and the number of eggs laid were monitored daily during their whole lives. Total and early fecundity were estimated as the number of eggs laid during the first 2 days and over the lifespan of individual B female, respectively. The wet body weight of one‐day‐old virgin adults was measured to the nearest 0.0001 g.

### 2.6 Wolbachia

Many insects are infected by maternally inherited, cytoplasmic bacteria such as *Wolbachia*, which can confound the results of experiments that aim to investigate mitochondrial effects on fitness. *Wolbachia* infections have been screened for in many *A. obtectus* populations, including lines used here, but have never been detected (see, e.g., Đorđević et al. 2017).

### 2.7 Statistical Analysis

Data on life history traits were subjected to mixed‐model one‐way ANOVAs, using Type III sum of squares (SAS 9.3, GLM procedure) (SAS Institute Inc. 2010). Within each sex, mitochondrial haplotype was used as a fixed factor, while mitochondrial replicas were modeled as a random factor nested within mtDNA haplotype. Body weight was used as a covariate in the analyses of early and total fecundity and lifespan. Prior to analyses, fertility and longevity data were log transformed, following an examination of the normality and homogeneity of variance assumptions. Multiple post hoc comparisons among pairs of means were performed using the Scheffé test. To test for independent mitochondrial effects among the sexes, Pearson phenotypic correlations between early fecundity and fertility of TFT males and life history of TFT females were calculated using SAS PROC CORR.

## 3 Results

**FIGURE 2:**
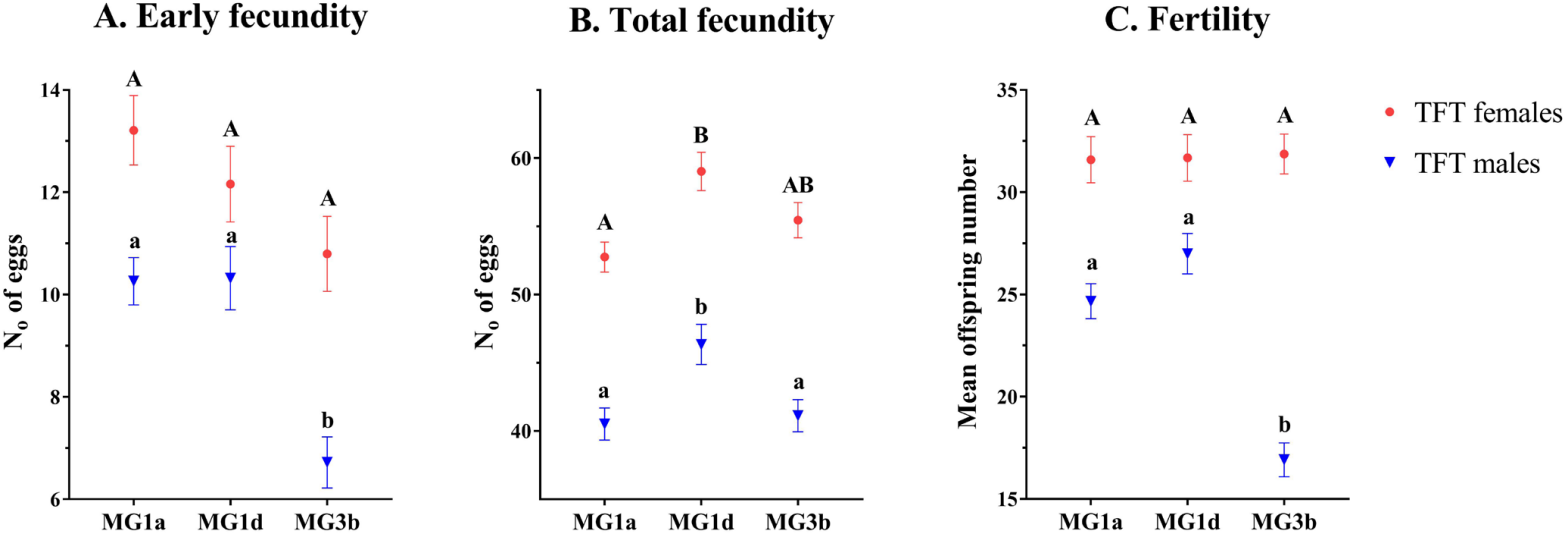
Mean values (±SE) of early fecundity (A), total fecundity (B), and fertility (C) of mated TFT females (red) and males (blue) harboring one of the three mtDNA haplotypes (MG1a, MG1d, or MG3b). Values denoted with capital letters (A and B) differ significantly between TFT females and those marked with lowercase letters (a and b) differ significantly between TFT males (Scheffé test; *p* < 0.05).

In seed beetles, the fertile ejaculate is the main trigger for oviposition (Huignard 1983). As an indicator of sperm quality, we tested how the TFT males carrying specific mitochondria affected the early fecundity of reference females. Our analyses revealed that females from Base (B) population mated to MG3b males postponed their egg laying, as evidenced by a 35% decrease in early fecundity compared to ones mated with MG1a and MG1d males (Figure 2A). However, the mitochondrial haplotype did not explain a significant proportion of the variance in this trait (*F* = 4.82, df = 2, 3.00, *p* = 0.12) due to the significant variance of replicas nested within each mitotype (*F* = 3.32, df = 3, 322, *p* = 0.02). This finding suggests significant genetic heterogeneity of the outbred B population that was used for the creation of TFT females and males. Another characteristic of the reproductive behavior of seed beetles is that females mated with subfertile males will lay both unfertilized and fertilized eggs until the end of their lifetime. This phenomenon is known as egg dumping. Total fecundity is therefore the sum of both fertilized and unfertilized eggs. Thus, it was not surprising that we found no significant difference between the total fecundity of B females regardless of the fertility status of TFT males carrying different mitochondria (Figure 2B; *F* = 0.55, df = 2, 3.03, *p* = 0.63). In addition, the variation between mt replicas was significant for this trait (*F* = 7.48, df = 3, 316, *p* < 0.0001). In TFT females, we found no effect of mitotype on early or total fecundity (Figure 2A,B; *F* = 0.42, df = 2, 3.03, *p* = 0.69 and *F* = 0.18, df = 2, 3.08, *p* = 0.84, respectively); however, high variation among mt replicas was detected (*F* = 8.16, df = 3, 344, *p* < 0.0001 and *F* = 3.19, df = 3, 342, *p* = 0.02, respectively). On average, the TFT females had higher mean values for early and total fecundity than the reference B females (Figure 2A,B). This is not surprising considering that the TFT females contain 50% of the L nuclear material, which has shown high reproductive output in our previous studies (Stojković et al. 2015).

TFT males carrying the mitochondrial haplotype MG3b had on average 34% lower fertility compared to the other mitotypes (Figure 2C). The general linear type III model revealed a significant effect of mitochondrial haplotype on fertility (*F* = 35.39, df = 2, 3.00, *p* = 0.008), while there was no variation between mt replicas (*F* = 1.02, df = 3, 333, *p* = 0.3849). We found no significant effect of the mitochondria on fertility variance in TFT females (Figure 2C; *F* = 0.15, df = 2, 3.00, *p* = 0.87). The variation among the mt replicas was low, but significant (*F* = 3.24, df = 3, 334, *p* = 0.02). These results, together with the early fecundity results, suggest that MG3b males have lower sperm quality compared to the other two mitotypes, while there is no negative effect on reproductive output in TFT females.

**FIGURE 3:**
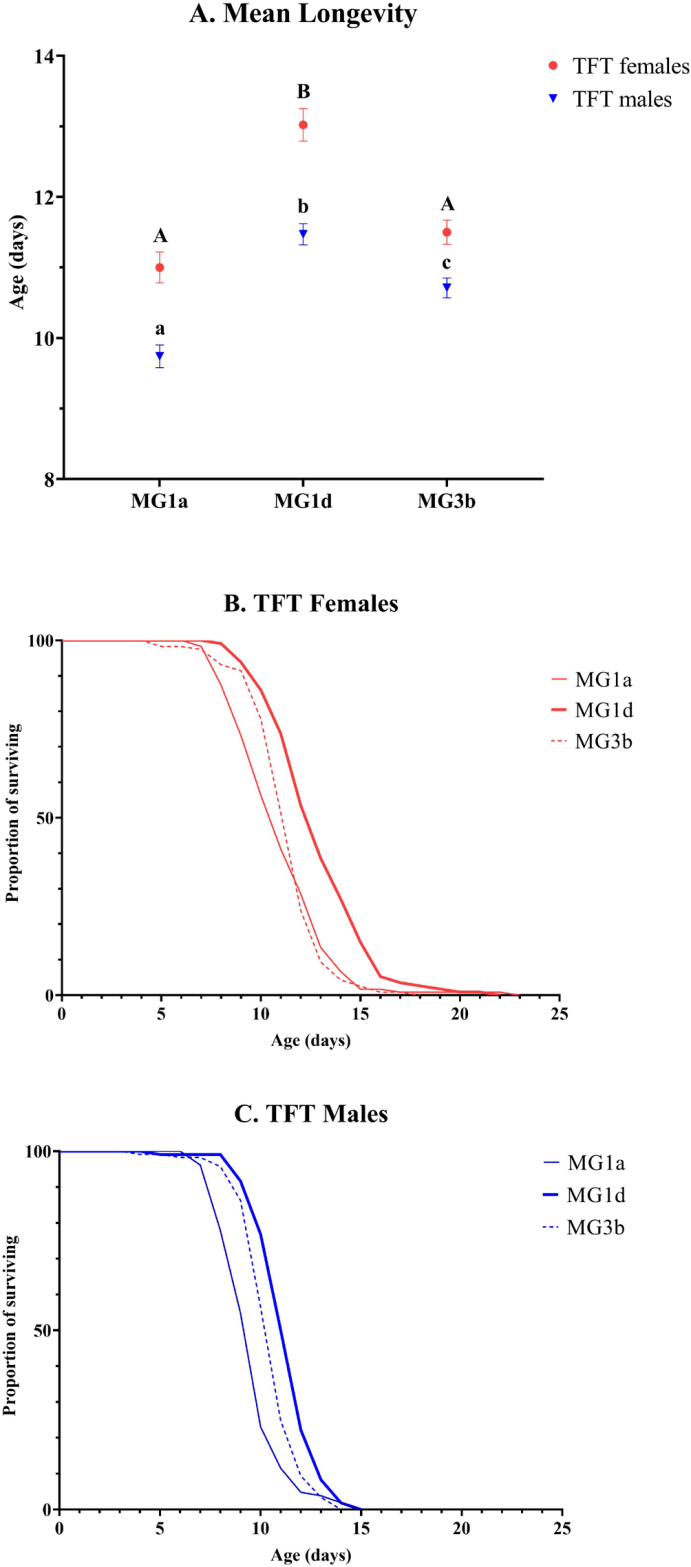
Mean (±SE) longevity (A) and survival curves of mated TFT females (B) and TFT males (C) harboring one of the three mtDNA haplotypes (MG1a, MG1d, or MG3b). Values denoted with capital letters (A and B) differ significantly between TFT females and those marked with lowercase letters (a, b, and c) differ significantly between TFT males (Scheffé test; *p* < 0.05). Thin lines represent MG1a, thick lines MG1d, and dashed lines MG3b beetles.

Comparison of longevity data showed that MG1d females and males lived on average 15% longer than MG1a beetles and 9% longer than MG3b beetles (Figure 3A). However, a one‐way ANCOVA for longevity with body weight as a covariate showed no significant effect of mitochondria in either sex (TFT females: *F* = 0.97, df = 2, 3.02, *p* = 0.47; TFT males: *F* = 2.11, df = 2, 3.02, *p* = 0.27). However, significant variance of replicas nested within each mitotype was detected in both sexes (*F* = 15.71, df = 3, 343, *p* < 0.0001 and *F* = 10.74, df = 3, 322, *p* < 0.0001, respectively). The survival curves of the TFT females and males from all three mitotypes (MG1a, MG1d, and MG3b) are shown in Figure 3B,C. In both sexes with MG3b mitotype, the survival curves are shifted between MG1a and MG1d, which is in concordance with the mean longevity data.

When combining genotyping of protein‐coding mtDNA genes with reproductive and life history effects, we could identify mutations in the MG3b mitotype as TFT candidate mutations (Table 2). We identify these mutations in the *cox2* gene at codon position 34, which causes the amino acid substitution of Ile (ATT) with Val (GTT), in *cytb* at codon position 334, which encodes the substitution of Ile (ATC) with Val (GTC), in *atp8* (Phe‐20‐Tyr), two *atp6* codons (Gln/Arg‐45‐Leu and Ser‐64‐Leu) and in two *nad2* codons (Ile‐73‐Thr and Val (GTA)‐138‐Met (ATA)).

Finally, we calculated the Pearson correlation coefficient between early fecundity of MG3b TFT males and early fecundity, fertility, and longevity of MG3b TFT females. Our analyses revealed no significant correlations between these traits (*r* = 0.14, *p* = 0.14; *r* = − 0.02, *p* = 0.84; *r* = 0.08, *p* = 0.39, respectively). Additionally, we tested for correlations, within the MG3b mitotype, between fertility of TFT males and early fecundity, fertility, and longevity of TFT females. Again, none of these correlations were significant (*r* = −0.06, *p* = 0.49; *r* = 0.11, *p* = 0.25; *r* = 0.03, *p* = 0.75, respectively).

## 4 Discussion

Our previous research on laboratory populations of the seed beetle *Acanthoscelides obtectus* has shown that the mitochondrial genome plays an important role in the evolution of life history traits. It was clearly demonstrated that changes in mtDNA variation could be expected as an outcome of natural selection depending on diverse environmental pressures (Đorđević et al. 2015; Stojković et al. 2017). As discussed in Stojković and Đorđević (2017), the link between the fitness components and the two genomes, that is, the mitochondrial and nuclear genomes, is rooted in their coevolved epistatic interactions that determine the amount of energy available for all biological functions. In a detailed study of the role of each ETC complex in modeling life history strategies, we constructed mitonuclear introgression lines of *A. obtectus* and confirmed that (i) ETC activity was significantly depressed when nuclear genomes were coexpressed with mitochondrial genomes from the opposing selection regime and (ii) many of the influences of mtDNA variation on life history traits were sex‐specific and largely compatible with the hypothesis that males represent a genetic dead‐end for mtDNA evolution. In other words, the negative effects of mtDNA variation were more pronounced in male than in female beetles (Đorđević et al. 2017).

Following these results, we further investigated the possibility that the specific mtDNA haplotype could serve as TFT candidate, that is, the carrier of Mother's Curse (MC) mutations that reduce male, but not female reproductive output and have limited or no positive pleiotropy in other life history traits within and between the sexes. The most pronounced result of this study is that we have found one such mtDNA haplotype—the MG3b. The non‐synonymous mutations in the subunits of complexes I, III, IV, and V (Table 2) were shown to have male‐specific effects on fertility and early fecundity of MG3b beetles. Considering the fact that ATP production and controlled ROS levels are required for proper sperm function (i.e., motility, capacitation and acrosome reaction), it is not surprising that mutations in the abovementioned complexes are associated with male subfertility. A previous study has shown that a non‐synonymous mutation in the mitochondrial cytochrome b subunit of the complex III (Ala‐278‐Thr) in *Drosophila melanogaster* from Brownsville, USA (BRO mitotype) reduces male reproductive capacity (Clancy, Hime, and Shirras 2011). The cellular mechanism of BRO infertility is a defect in sperm maturation, which is probably due to elevated ROS generation by CIII (Clancy, Hime, and Shirras 2011). In a more recent study, another naturally occurring missense mtDNA mutation in cytochrome c oxidase subunit II (Gly‐177‐Ser) was reported, which leads to male subfertility, but has no effect on females (Patel et al. 2016). The decreased fertility of CII^G117S^ flies was due to reduced sperm motility and improper maturation. This defect in sperm development and function was linked with impaired complex IV activity and reduced ROS levels, but not ATP production (Patel et al. 2016). Interestingly, both complexes which were previously found to contain MC mutations and reduce male fertility, also harbor non‐synonymous mutations in MG3b mitotype of seed beetles. This could explain the mechanisms of the consequential male subfertility in MG3b *A. obtectus* .

The second criterion for the TFT candidate mutation has also been confirmed for the MG3b mitotype. This mtDNA haplotype had no effect on longevity in either females or males of the seed beetle, which is important for the success and applicability of TFT. Generally, our results suggest that the sex‐specific effects of mitotypes in seed beetles are stronger for reproductive function than for the maintenance of somatic lifespan, which is largely shared between the sexes (Turelli and Orr 2000). A study by Immonen et al. (2016) on the seed beetle *C. maculatus* , also found no sex differences in mitochondrial effect on longevity. However, other studies on longevity have reported either male‐biased mitochondrial effects (Camus, Clancy, and Dowling 2012; Aw et al. 2017) or mtDNA effects that are stronger in females (Nagarajan‐Radha et al. 2019; Watson et al. 2022). Discrepancies in MC effects exist even within the same taxa, such as those found in *Drosophila*. For example, Camus, Clancy, and Dowling (2012) screened the panel of 13 mitotypes and discovered male biases in longevity and aging rates in contrast to Nagarajan‐Radha et al. (2019) study where the effects of mitochondrial haplotype variation on longevity were larger in females. The inconsistency in the results of the two studies could be directly attributable to differences in diets since the mitochondrial effects are highly dependent on the environment. Overall, studies investigating the effects of MC mutations on lifespan are still scarce, and evidence for such effects is mixed and limited to only a few taxa (Edmands 2024). However, in a recent broad taxonomic study that included a survey of 108 mammalian species, no sex‐specific differences were found in the impact of mtDNA mutation accumulation on lifespan and aging rate (Cayuela et al. 2023).

The fact that we found no evidence of a negative intersexual correlation between the reproductive output of TFT males and the early fecundity, fertility, and longevity of TFT females suggests that the MG3b mitotype has spread in a population by random drift (evidence of the weak form of the MC hypothesis), rather than by positive selection in females (strong form of the MC hypothesis, Dowling and Adrian 2019). This result is not surprising given that the theory suggests that MC mutations are much more prevailing in invertebrate systems with high dispersal abilities, large effective population sizes and relatively simple social behaviors (Smith and Connallon 2017). The population and biological characteristics of our model system *A. obtectus* fall under all of the above criteria. Additionally, the polyandrous mating system of *A. obtectus* , which increases the levels of random mating and outbreeding, limits the probability of MC females mating with their subfertile brothers (Zhang, Guillaume, and Engelstädter 2012). Consequently, this reduces the effects of inbreeding and kin selection, and elevates the accumulation of male‐harming mutations in the mitochondrial genome in a population (Hedrick 2012; Unckless and Herren 2009; Wade and Brandvain 2009; Zhang, Guillaume, and Engelstädter 2012). Outbreeding can even lead to weak positive selection for MC mutations, as males carrying the mutation reduce the fitness of wild‐type females more than that of MC females (Engelstädter and Charlat 2006). In this context, pest species can serve as ideal empirical systems for identifying mtDNA loci that disproportionately influence the variance in male fitness.

The Trojan Female Technique achieves sustainable, self‐perpetuating population control by releasing females carrying naturally occurring mitochondrial MC mutations that negatively affect male but not female fertility. TFT is thus a promising, species‐specific, reversible, and humane form of target population suppression. Modeling studies suggest that both single large releases (10% of the population) and relatively few small repeated releases (1% of the population) of TFT females provide effective and persistent control within relatively few generations (Gemmell et al. 2013). Recent work in fruit flies has shown that the BRO mitotype can reduce population size by an average of 8% over 10 generations at a haplotype frequency of 75% at the start of treatment (Wolff et al. 2017). The 34% reduction in fertility of the MG3b mitotype demonstrated in our study is well within the range of effect sizes predicted for the TFT approach for biological control (Wolff et al. 2017). However, there are many factors that could influence the efficacy of TFT, such as reduced mating competitiveness of TFT males or general effects of MC mutations on male fertility in a range of nuclear genomic and environmental contexts. Thus, it remains to be tested whether the effects of MG3b will be upheld in different nuclear backgrounds or environmental conditions. Therefore, we point out that while the strength of our experimental design is that we were able to assess sex‐specific effect of specific mitotypes on reproductive output, a limitation of our study is that these effects were all assessed within a single outbred nuclear genetic background and in benign laboratory conditions.

Our findings have implications for future biocontrol studies in this and other insect pests by investigating whether the release of multiple TFT mitotypes can suppress target populations. In addition, TFT could be used in combination with SIT and/or the incompatible insect technique to improve efficiency and increase the range of pests targeted by environmentally friendly pest control strategies. Finally, understanding the effects of MC mutations and interactions with their coevolving nuclear genes is not only crucial for the development of new biocontrol techniques and strategies, but could also have very practical implications for conservation breeding programs and reproductive medicine (Iverson 2024; Leeflang, Van Dongen, and Helsen 2022).

## Conflicts of Interest

The authors declare no conflicts of interest.

## Supporting information


**Table S1.** Details on primers and marker genes used in analysis of *Acanthoscelides obtectus* mtDNK variability.

## Data Availability

The dataset associated with this publication is archived and publicly available in RADaR—the Digital Repository of Archived Publications, Institute for Biological Research “Siniša Stanković” at https://hdl.handle.net/21.15107/rcub_ibiss_6696.
