# Supplementary material for: Toward the Development of the Trojan Female Technique in Pest Insects: Male‐Specific Influence of Mitochondrial Haplotype on Reproductive Output in the Seed Beetle Acanthoscelides obtectus
Source: Evol Appl. 2024 Dec 26;17(12):e70065. doi: 10.1111/eva.70065 (PMC11671214; doi:10.1111/eva.70065)
Supplement: Supplementary file 1 — Table S1. Details on primers and marker genes used in analysis of Acanthoscelides obtectus mtDNK variability. [file EVA-17-e70065-s001.docx]

Supplementary Table S1.Details on primers and marker genes used in analysis of *Acanthoscelides obtectus* mtDNK variability

| **MtDNA amplification target** | | **Primer name** | **Primer sequence 5' → 3'** | **Target gene length (base pair - bp)** | |
| --- | --- | --- | --- | --- | --- |
| **Gene** | **Protein** |  |  | **PCR amplicon** | **Sequence** |
| *cox1* | cytochrome c oxidase subunit 1 | LCO1490-Ao F | TCTCTACGAATCATAAAGATATTGG | 709 bp | 657 bp |
|  |  | HCO2198-Ao R | TAAACTTCAGGGTGGCCAAAAAATCA |  |  |
| *cox3* | cytochrome c oxidase subunit 3 | C3-J4792-Ao F | TAGATGTAAGACCCTGACC | 706 bp | 621 bp |
|  |  | C3-N5460-Ao R | TCTACAAAATGYCAGTATCA |  |  |
| *nad4* | NADH dehydrogenase subunit 4 | N4-J8641-Ao F | CCTGATGAACACAAACCATG | 545 bp | 474 bp |
|  |  | N4-N9153-Ao R | TGAGGCTATCAACCAGAGCG |  |  |
| *cytb* | cytochrome b | CB-J10933-Ao F | GTTCTCCCCTGAGGGCAAATATC | 639 bp | 576 bp |
|  |  | CB-N11526-Ao R | TTCTACAGGGCGTGCTCCAATTCA |  |  |
| *nad1* | NADH dehydrogenase subunit 1 | N1-J11876-Ao F | CGAGGTAAAGTTCCCCGAACCCA | 767 bp | 678 bp |
|  |  | N1-N12595-Ao R | GTGGCCTTTTTGACTTTATTAGAGCG |  |  |
| *nad4l* & *nad6* | NADH dehydrogenase subunit 4L & NADH dehydrogenase subunit 6 | N4L-J9648-Ao F | ACCTAGTACACCCTCACAAAC | 972 bp | 153 bp *nad4l* & 509 bp *nad6* |
|  |  | CB-N10608-Ao R | CCTAAAAGGGATCCAAAATTTCA |  |  |
| *cox2* | cytochrome c oxidase subunit 2 | TL2-J-3033-Ao F | TCTAATATGGCAGAGTAGTGCA | 784 bp | 688 bp |
|  |  | TK-N-3782-Ao R | AAGTTCATTGCTTACTTTCAGCCATCT |  |  |
| *nad2* | NADH dehydrogenase subunit 2 | TM-J210-Ao F | AATAAAGCTATTGGGTTCATACCC | 829 bp | 657 bp |
|  |  | N2-N993-Ao R | GGAAGAAATCCTAAGAAAGGAGG |  |  |
| *nad3* | NADH dehydrogenase subunit 3 | C3-J5470-Ao F | GCTGCCGCTTGATACTGACA | 753 bp | 354 bp |
|  |  | TN-N6160-Ao R | TCAATTTGATCATTAACAGTGA |  |  |
| *nad5* | NADH dehydrogenase subunit 5 | N5-J7077-Ao F | CTAAATCTTTAGAATAAAACCC | 754 bp | 668 bp |
|  |  | N5-N7793-Ao R | TTAGGCTGAGATGGTTTAGG |  |  |
| *atp8* & *atp6* | ATP synthase F0 subunit 8 & ATP synthase F0 subunit 6 | TK-J3790-Ao F | CATTAGATGGCTGAAAGTAAGCA | 795 bp | 156 bp *atp8* &  488 bp *atp6* |
|  |  | A6-N4552-Ao R | ATGGCCAGCGATTATATTAGC |  |  |
